# Spina-Bifida Cured by Injections of Iodine

**Published:** 1849-07

**Authors:** 


### SELECTIONS

FROM

### AMERICAN AND FOREIGN JOURNALS.

---

*Spina-Bifida Cured by Injections of Iodine.*—An Indiana journal reports a case of spina-bifida in a young lady, aged 13 years, cured by Dr. Brainard by the use of two small iodine injections practiced at an interval of twenty-five days from each other, and followed by compression. The tumor was situated upon the sacrum, was nine inches in circumference, and three inches in height, and accompanied by paraplegia and recto-vesical incontinence. The injection, composed of one grain of iodide of potassium, half a grain of iodine, and one drachm of distilled water, was introduced by the subcutaneous method, and allowed to remain in the tumor. The reaction was considerable after the first injection, but quite moderate after the second. Compression obliterated the sac. Radical cure in two months. Amelioriation of the paraplegia. In very young children and infants a solution of half the strength of the above.—*Annales de Therapeutique* —D. W. Y.

